# Design of RNAi Hairpins for Mutation-Specific Silencing of Ataxin-7 and Correction of a SCA7 Phenotype

**DOI:** 10.1371/journal.pone.0007232

**Published:** 2009-09-30

**Authors:** Janine Scholefield, L. Jacquie Greenberg, Marc S. Weinberg, Patrick B. Arbuthnot, Amr Abdelgany, Matthew J. A. Wood

**Affiliations:** 1 Division of Human Genetics/MRC/UCT Human Genetics Research Unit, Institute of Infectious Disease and Molecular Medicine, Faculty of Health Sciences, University of Cape Town, Cape Town, South Africa; 2 Department of Molecular Medicine and Haematology, University of Witwatersrand, Johannesburg, South Africa; 3 Department of Physiology, Anatomy and Genetics, University of Oxford, Oxford, United Kingdom; National Institutes of Health, United States of America

## Abstract

Spinocerebellar ataxia type 7 is a polyglutamine disorder caused by an expanded CAG repeat mutation that results in neurodegeneration. Since no treatment exists for this chronic disease, novel therapies such post-transcriptional RNA interference-based gene silencing are under investigation, in particular those that might enable constitutive and tissue-specific silencing, such as expressed hairpins. Given that this method of silencing can be abolished by the presence of nucleotide mismatches against the target RNA, we sought to identify expressed RNA hairpins selective for silencing the mutant *ataxin-7* transcript using a linked SNP. By targeting both short and full-length tagged *ataxin-7* sequences, we show that mutation-specific selectivity can be obtained with single nucleotide mismatches to the wild-type RNA target incorporated 3′ to the centre of the active strand of short hairpin RNAs. The activity of the most effective short hairpin RNA incorporating the nucleotide mismatch at position 16 was further studied in a heterozygous *ataxin-7* disease model, demonstrating significantly reduced levels of toxic mutant ataxin-7 protein with decreased mutant protein aggregation and retention of normal wild-type protein in a non-aggregated diffuse cellular distribution. Allele-specific mutant ataxin7 silencing was also obtained with the use of primary microRNA mimics, the most highly effective construct also harbouring the single nucleotide mismatch at position 16, corroborating our earlier findings. Our data provide understanding of RNA interference guide strand anatomy optimised for the allele-specific silencing of a polyglutamine mutation linked SNP and give a basis for the use of allele-specific RNA interference as a viable therapeutic approach for spinocerebellar ataxia 7.

## Introduction

Spinocerebellar ataxia 7 (SCA7) is a late onset neurodegenerative disease which presents with a classic autosomal dominant ataxia but which is uniquely associated with macular degeneration [1]. It is part of a group of nine known polyglutamine (polyQ) disorders which share expanded (CAG) repeat mutations that translate into polyQ tracts [2]. While it is considered one of the rarer dominant inherited ataxias, in South Africa it is the second most prevalent of the autosomal dominant cerebellar ataxias [3]. Removal of accumulated mutant polyQ proteins has been shown to ameliorate the disease phenotype in mouse [4] and drosophila models [5], which is consistent with a toxic gain-of-function disease mechanism [6]. However, some studies report that wild-type protein function may be affected by the presence of the mutant resulting in an additional loss-of-function mechanism contributing to disease pathogenesis [7]–[9]. Most RNA interference (RNAi)-based therapeutic approaches to suppress the expression of toxic polyQ proteins have been demonstrated in mouse models of polyQ disorders, including SCA1, SCA3 and HD [10]–[12]. While important, these studies have targeted suppression of the human disease transgene in the mouse and therefore have not investigated the effects of concomitant wild-type allele suppression or investigated the mechanistic basis for specific suppression of polyQ disease alleles.

The sequence-specific nature of post-transcriptional RNAi has therapeutic potential where selective inhibition of a mutant transcript would leave the wild-type intact for normal cellular function [13], [14]. This can be achieved by exploiting an endogenous single base pair difference between the wild-type and mutant transcripts, such that an effective RNAi sequence can be designed with complete homology to the mutant transcript but containing a single nucleotide mismatch with respect to the wild-type. Effective double-stranded RNAi sequences, whether small interfering RNAs (siRNAs) or derived from expression of hairpin RNAs (shRNAs), undergo strand selection to generate an active single-stranded guide sequence incorporated into the RISC complex. This 19–24 nucleotide guide strand comprises functional domains, including the seed region nucleotides 2–8 for target recognition and central nucleotides 10 and 11 for Ago2-mediated cleavage. An important question therefore is which guide strand positions are least tolerant of the presence of a destabilising nucleotide mismatch and therefore how guide strand anatomy can be optimised for mutation-specific silencing

Previous studies have shown degrees of target discrimination with the single nucleotide mismatch incorporated at central guide strand positions [15], [16]. Subsequently, using an extensive panel of siRNAs, Schwarz and colleagues demonstrated that mismatches 3′ to the centre of the guide strand had increased selectivity against huntingtin and SOD1 targets [17]. Furthermore, this study showed that purine:purine nucleotide mismatches resulted in maximum selectivity, whereas purine:pyrimidine and in particular the weak G:U mismatch (in which a single hydrogen bond is formed between the two nucleotides) yielded significantly less discrimination, a finding suggested in earlier studies [18]–[21]. Other investigations have used the mutation itself to discriminate between mutant and wild-type transcripts [22], [23], and although structural differences between polyQ transcripts may make this a feasible approach in theory [24] it is not ideal given the potential off-target effects of a CAG repeat sequence [14], [25]. However, a common SNP (located in the 3′ region of *atxn7, NM000333.3*) linked to the SCA7 mutation has been identified, such that over 50% of South African SCA7 patients have the ‘A’ and ‘G’ alleles associated with the mutant and wild-type transcripts respectively [26]. Targeting of this SNP allows detailed study of how guide strand anatomy can be optimised for single nucleotide specific silencing and whether this could form the basis of a potential therapy for SCA7.

## Results

### shRNAs targeting ataxin-7 with effective single nucleotide discrimination

**Figure 1:**
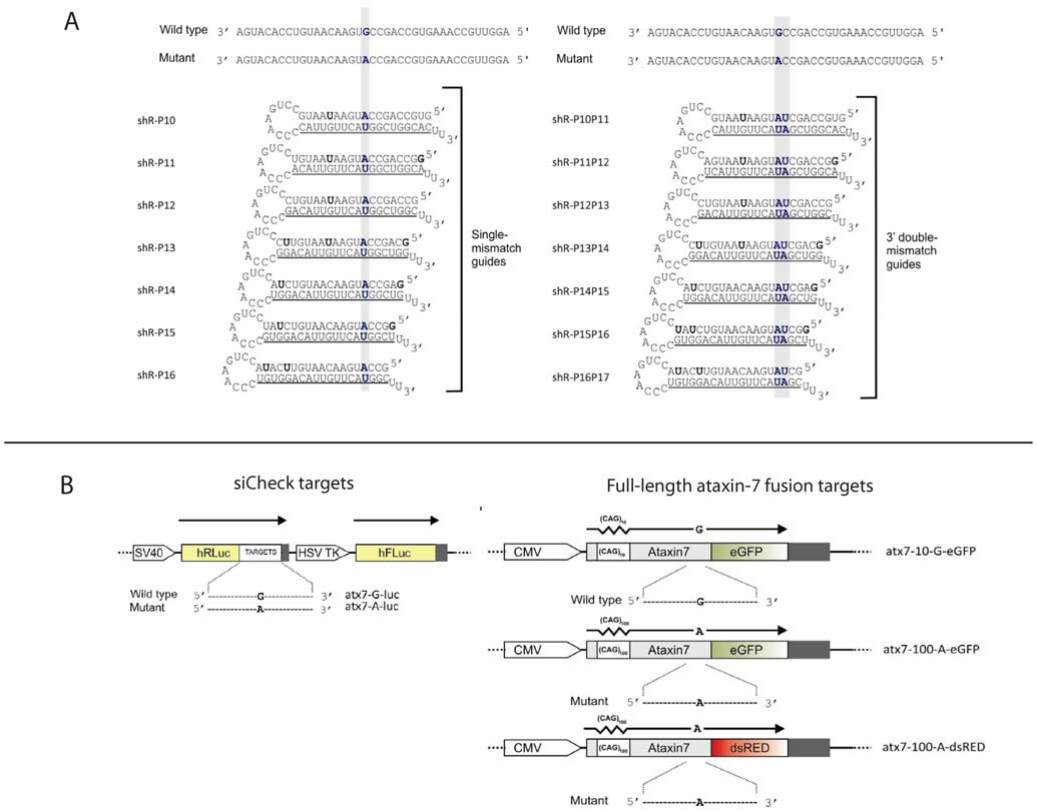
RNAi-based shRNAs and target vectors designed for *atxn7* knockdown. (A) Convention for the diagram follows Schwarz and colleagues (16). Sequences of expected single and double mismatch guide strands (underlined) processed from an shRNA format indicating the site of the primary mismatch to the G allele of the wild-type *atxn7* target. Sequence context of both the wild-type and mutant targets are shown. Functional asymmetry was induced by creating G:U mis-pairings in the 3′ end of the anti-guide strand; these are highlighted in bold. The shRNAs were labelled according to the position of the primary mismatch relative to the 5′ end of the guide strand; followed by any additional mismatches. The nomenclature is based on a 21bp guide strand after cleavage of the anti-sense strand by Dicer. (B) Schematic representations of the fused target-reporter cassettes. atx7-G-luc and atx7-A-luc are the wild-type and mutant reporter siCHECK plasmids used in the luciferase assay, and included a short region of 60 bp of *atxn7* target sequence spanning the SNP fused to the *Renilla* luciferase reporter gene. atx7-10-G-eGFP, atx7-100-A-eGFP, atx7-100-A-DsRED are the 3 reporter plasmids used in the full-length hemi- and heterozygous assays and consist of the full-length *atxn7* cDNA. The wild-type construct (atx7-10-G-eGFP) has a CAG repeat length of 10 incorporating the G allele of the SNP, fused to eGFP. The two mutant constructs (atx7-100-A-eGFP, atx7-100-A-DsRED) have CAG repeat lengths of 100, as well as the A allele of the SNP, and are fused to eGFP and DsRED respectively.

**Figure 2:**
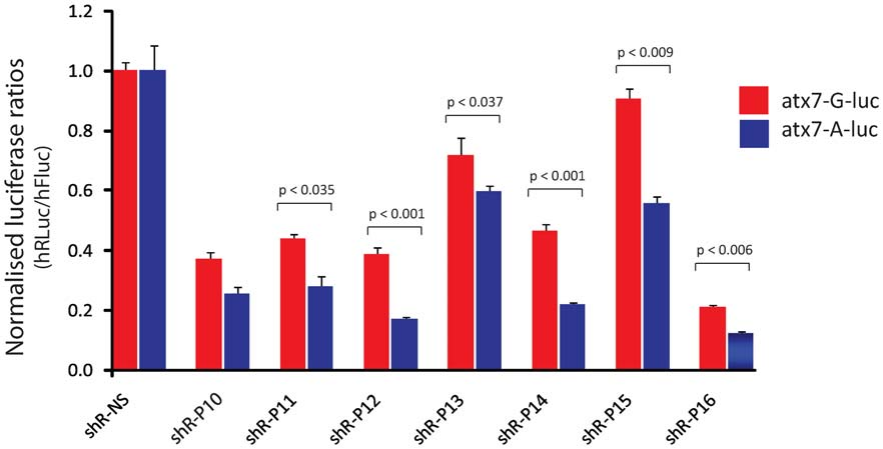
Analysis of series of single mismatched shRNA guide sequences targeting the G>A SNP in *atxn7* in a dual luciferase assay. Relative levels of R*enilla* luciferase (hRluc) expression normalized to firefly luciferase (hFluc) expression for single mismatches (guide sequences shown in Figure 1A). Each experiment was performed in triplicate and the data is relative to that measured using a non-specific shRNA, shR-NS. Average±standard deviation is shown. Statistically significant differences (p<0.05) between wild-type and mutant silencing are indicated by corresponding p values. Relative expression of wild-type and mutant targets is represented by red and blue bars respectively.

In order to identify effective post-transcriptional RNAi sequences targeting the *atxn7* linked SNP, multiple short hairpin RNAs (shRNAs) were screened, each incorporating the weak G:U mismatch created by the targeted SNP with respect to the wild-type transcript successively at positions 10 to 16 from the 5′ end of the active shRNA guide sequence (Figure 1A). These shRNAs were screened in an assay in which a short 60 bp target of either the mutant or the wild-type ataxin7 gene sequence was inserted in the 3′UTR of Renilla luciferase and normalised to background Firefly luciferase (Figure 1B). Nucleotide mismatches incorporated at shRNA positions 11-16 all showed effective discrimination between the wild-type and mutant targets (Figure 2A), shR-P15 demonstrating the greatest discrimination with the wild-type target minimally affected retaining 90% expression and the mutant knocked down to nearly 50% relative to a non-specific shRNA control (p<0.05). However, overall target knockdown was less efficient than for shR-P12 (wild-type and mutant targets knocked down to 40% and 20% respectively) and shR-P14 (wild-type and mutant targets knocked down to 45% and 20% respectively). Interestingly, the shR-P16 construct showed little mutant-specific discrimination, knocking down both wild-type and mutant targets with high efficiency (20% and 10% remaining of the wild-type and mutant targets), and indicating high tolerance of the single nucleotide mismatch incorporated at this predicted shRNA guide strand position. Given the weak nature of the *atxn7* G:U nucleotide mismatch, we investigated whether enhanced wild-type:mutant discrimination could be achieved by incorporating a range of secondary mismatches in addition to the primary nucleotide mismatch (Figure 1A), however improved mutant selectivity was not obtained (Figure S1A). It was noted that the inclusion of secondary mismatches resulted in decreased levels of knockdown at almost all positions tested indicating that the secondary mismatch ablated efficient RNAi against both targets.

### Effective shRNA-mediated silencing of full-length mutant atxn7

**Figure 3:**
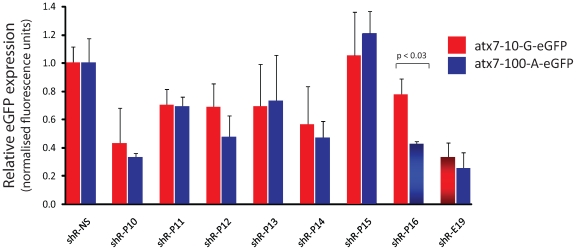
Single mismatched shRNAs targeting the *atxn7* G>A SNP in a full-length hemizygous assay. Quantitation of fluorescence in HEK293 cells transfected with wild-type (atx7-10-G-eGFP) or mutant (atx7-100-A-eGFP) expression plasmids and the indicated shRNA is shown. Each experiment was performed in triplicate and the data is relative to that measured using a non-specific shRNA, NS. The shR-E19 hairpin was pE19; a published U6/shRNA expression plasmid targeting eGFP (37). Average±standard deviation is shown. Statistically significant differences (p<0.05) between wild-type and mutant silencing are indicated by corresponding p values. Wild-type and mutant targets are represented by red and blue bars respectively.

Given that the initial data shown (Figures 2 and Figure S1A) and that obtained by others [17] was acquired from screens of short target mRNA sequences, we sought to model the endogenous SCA7 disease more closely by studying shRNAs targeting the full-length endogenous *atxn7* cDNA transcript (Figure 3). This assay was validated using an shRNA specific for the GFP sequence (shR-E19) which consistently showed 80% silencing efficiency. Using a full length *atxn7* target transcript fused to an eGFP reporter target, knockdown was found to be reduced across the panel of shRNAs studied (Figure 3). Again, shRNAs incorporating the single nucleotide mismatches at position 12 showed some mutant selectivity (*wild-type and mutant targets knocked down to 70% and 50% respectively*), however shR-P15 – the most discriminatory shRNA in the dual luciferase assay against the short target transcript – showed no ability to knockdown either the full-length mutant or wild-type *atxn7* gene (Figure 3). Interestingly, shR-P16, which showed the most effective knockdown against both short targets in the luciferase assay, demonstrated the greatest selectivity against the full-length mutant target with 90% of wild-type and 50% of mutant expression remaining (p<0.05). Again, none of the double nucleotide mismatched shRNA hairpins showed any significant mutant selectivity against the full-length *atxn7* target (Figure S1B). The selective shR-P16 was further studied by testing whether enhanced discrimination was possible by incorporating further modifications within this shRNA guide strand, including shifting the position of the secondary mismatch and the inclusion of additional mismatches (Figure S2A). None of the further modified position 16-based shRNA sequences showed either significant knockdown or improved mutation-specific selectivity (Figure S2B), and therefore further analysis of only the most efficacious shR-P16 construct was undertaken.

### Effective silencing of mutant atxn7 in a heterozygous SCA7 disease model

**Figure 4:**
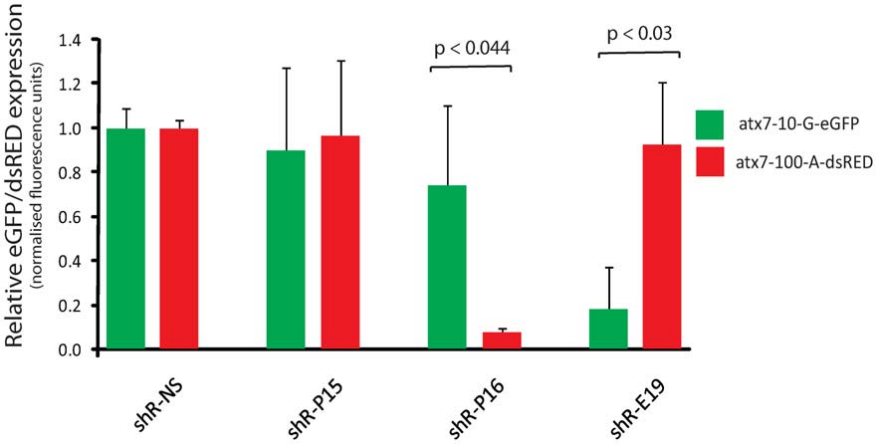
Knockdown of targets in a heterozygous assay using shRNAs. Quantification of fluorescence in HEK293 cells co-transfected with wild-type atx7-10-G-eGFP and mutant atx7-100-A-DsRED expression plasmids and the indicated shRNA is shown. Each experiment was performed in triplicate and the data is relative to that measured using a non-specific shRNA, NS. Average±standard deviation is shown. The shR-E19 hairpin was pE19; a published U6/shRNA expression plasmid targeting eGFP (37). Statistically significant differences (p<0.05) between wild-type and mutant silencing are indicated by using the one-tailed t-test. Wild-type and mutant targets are represented by green and red bars respectively.

To more closely mimic the endogenous SCA7 disease state, a heterozygous model was established in which both full-length wild-type and mutant *atxn7* cDNA expression plasmids were co-transfected into HEK293 cells (Figure 1B). shR-P16, which previously demonstrated high efficiency target selection in the full-length hemizygous assay, again showed high mutation-specific selectivity in the heterozygous disease model system, efficiently knocking down the mutant transcript to 7% of control levels (p<0.05) whilst minimally affecting the wild-type (74% of the control levels; Figure 4). Note that the GFP specific shRNA (shR-E19) control showed specificity for the GFP tagged wild-type transcript and not the dsRED tagged mutant transcript.

To exclude the possibility that sequence specific effects of shR-P16 were influenced by the particular reporter tag used, experiments were conducted utilizing the dsRED tag in a hemizygous assay. Silencing levels of 30% were obtained (Figure S4) which are similar to that obtained with the GFP tagged transcript, confirming that the sequence specificity of shR-P16 was independent of the reporter tag used (Figure 3). Moreover, the sequence specificity of the shR-P16 was not significantly affected by a change in the number of repeats; the G allele of the SNP being resistant to silencing in the presence of either 10 or 100 CAG repeats (Figure 3 and Figure S4).

### shRNA P16 targeting mutant atxn7 corrects the SCA7 cellular phenotype

**Figure 5:**
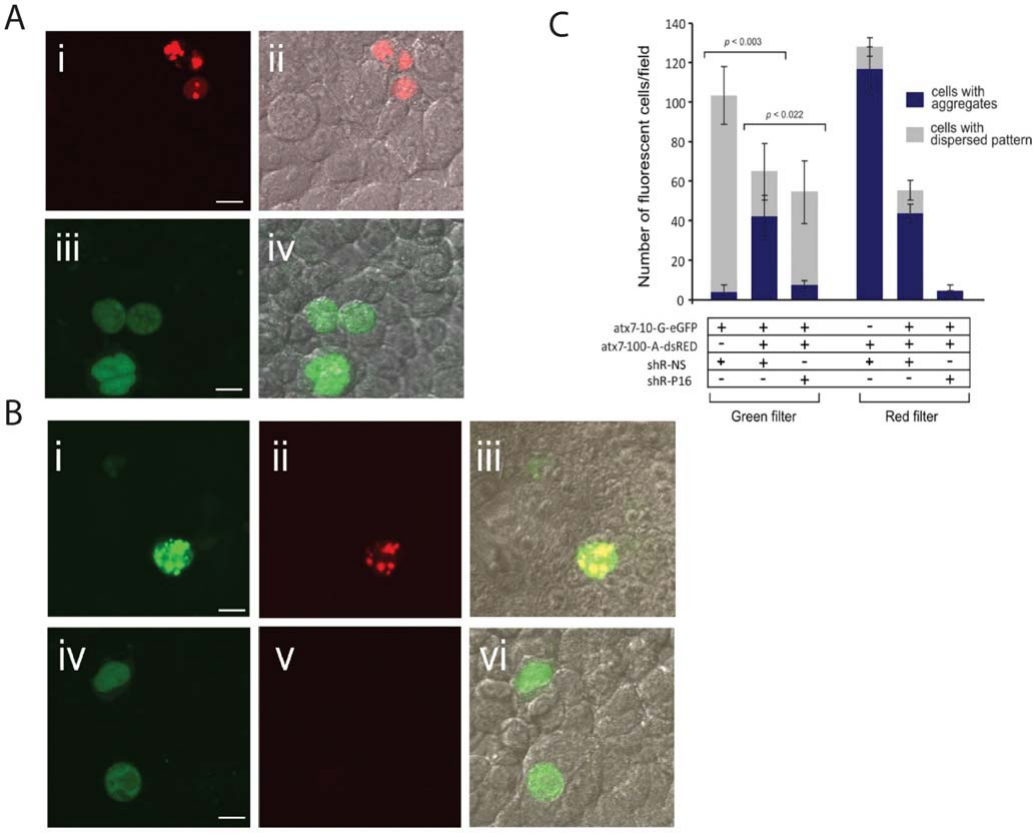
Investigation of aggregate formation. (A) Representative confocal images of cells transfected with (i, ii) mutant (atx7-100-A-DsRED) alone; (iii, iv) wild-type (atx7-10-G-eGFP) alone. (i) Image visualized under red fluorescence, (ii) red fluorescence merged with the bright field, (iii) green fluorescence, and (iv) green fluorescence merged with the bright field. (B) Representative confocal images of cells co-transfected with wild-type and mutant expression plasmids in addition to (i–iii) shR-NS or (iv–vi) shR-P16. (i) and (iv) show images under green fluorescence to reveal wild-type protein, (ii) and (v) show images under red fluorescence to reveal mutant protein, and (iii) and (vi) show images then merged under green and red fluorescence and bright field. Scale bar represents 10 µm. C. Cells expressing eGFP and/or DsRED were counted according to whether they contained aggregates or a dispersed pattern of expression of mutant and wild-type ataxin-7. Cells were transfected with wild-type target (atx-10-G-eGFP) alone; mutant target (atx7-100-A-DsRED) alone; mutant (atx7-100-A-DsRED), wild-type (atx-10-G-eGFP) and shR-NS (non-specific shRNA); mutant, wild-type and shR-P16. Cells were counted separately in the red and the green filter by collecting 3 representative images from each well and combining the total number. This was performed for each indicated combination in biological triplicate, yielding standard deviations. The bars comprise the total number of cells counted in each transfection; separated according to whether they contained aggregates (blue) or a dispersed pattern of expression (grey). Note that the decrease in expression from target vectors transfected alone to co-transfected cells is due to a promoter occlusion effect of co-expression of targets and not due to the addition of shR-NS which has no effect upon the target vectors (data not shown). Statistically significant differences in % of aggregate containing cells are indicated by corresponding p values.

It is well documented that mutant polyQ proteins form intracellular aggregates which are associated with, if not directly causative of, disease pathogenesis [27]. In addition, it has been shown that mutant ATXN7 protein recruits wild-type ATXN7 into intracellular inclusion-like aggregates [28]. In our studies, confocal microscopy showed that while the mutant protein (CAG_100_) forms large distinct aggregates, expression of the wild-type protein (CAG_10_) alone resulted in minimal aggregate formation (Figure 5A). However, heterozygous co-expression of both wild-type and mutant *atxn7* genes reproduces previous reports [28] in which the wild-type ATXN7 protein is found to co-localize with aggregates of mutant protein (Figure 5Bi–iii). Transfection of mutation-specific shR-P16 into these cells resulted not only in decreased numbers of cells with mutant protein aggregates, but also in wild-type protein normalised to a non-aggregated, more diffuse pattern of cellular localisation seen when the wild-type is transfected alone and typical of the non-disease state (Figure 5Biv–vi). To quantify these findings, the number of cells containing detectable aggregates or showing a dispersed pattern of wild-type protein localisation was determined following shR-P16 treatment and compared to that seen with the non-specific control (Figure 5C). This data shows a highly significant decrease in the number of cells expressing mutant protein aggregates in the presence of shR-P16, corroborating data from the fluorescent heterozygous assay. More importantly, these results further support the original observation that, not only does expression of both mutant and wild-type protein result in a significantly increased percentage of aggregates containing wild-type protein (p<0.05), but that treatment with shR-P16 promotes restoration of the wild-type protein expression pattern.

### Effective mutation-specific silencing with a primary microRNA mimic

**Figure 6:**
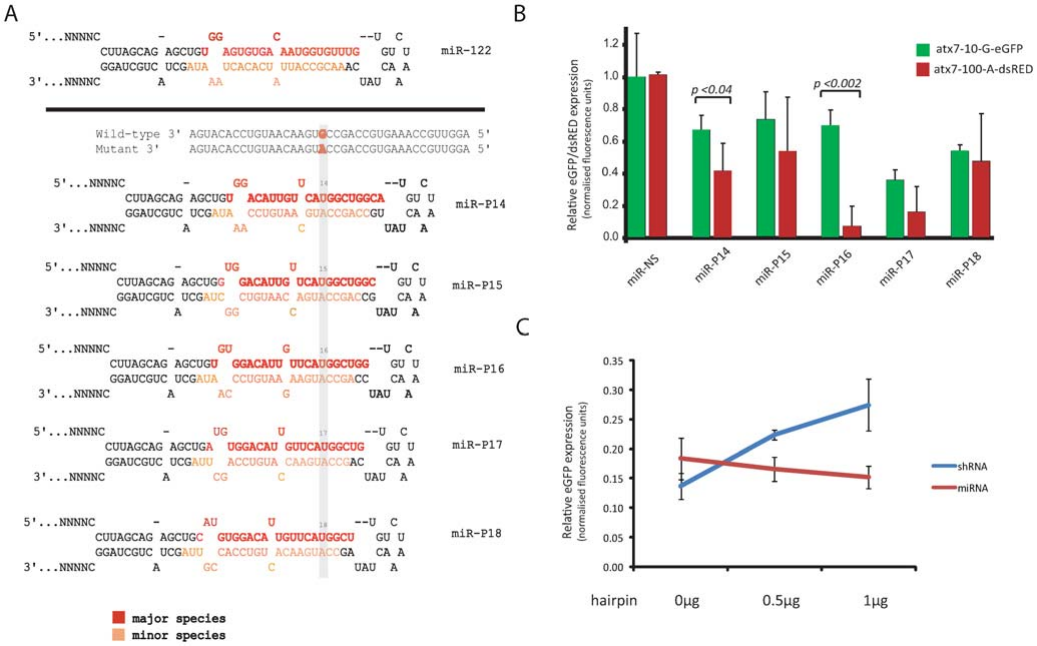
Knockdown of targets using pri-miRNA-based hairpins. A) Guide strands in pri-miRNA format, with sequences of mismatch guide strands processed from a miRNA format indicating the site of the primary mismatch to the G allele of the wild-type *atxn7* target. Sequence context of both the wild-type and mutant targets are shown. The miRNAs were labelled according to the position of the primary mismatch relative to the 5′ end of the guide strand. Major species refers to the strand most likely to be incorporated by RISC, while the minor species indicates the strand less likely to be incorporated. B) Knockdown of targets in a heterozygous assay using miRNAs. Quantification of fluorescence in HEK293 cells co-transfected with wild-type atx7-10-G-eGFP and mutant atx7-100-A-DsRED expression plasmids and the indicated miRNA. The data is relative to that measured using a non-specific miRNA, NS. Average±standard deviation is shown. Statistically significant differences (p<0.05) between wild-type and mutant silencing are indicated by using the one-tailed t-test. Wild-type and mutant targets are represented by green and red bars respectively. C. Increased addition of an shRNA but not a miRNA hairpin interferes with post-transcriptional gene silencing machinery. Low levels of a GFP target plasmid and GFP specific hairpin result in knockdown of GFP relative to a non-specific hairpin in the presence of 1 µg of empty vector, either pU6+1 or pCI_neo (vectors which contain the promoters used to transcribe shRNA and miRNA hairpins respectively). The shRNA data is relative to that measured using a non-specific shRNA, NS, while the miRNA data is relative to that measured using a non-specific miRNA, NS. The shR-E19 hairpin was pE19; a published U6/shRNA expression plasmid targeting GFP (37). Average±standard deviation is shown. Each experiment was performed in triplicate.

Primary miRNA mimics have advantages as gene silencing agents since they permit tissue specific regulation and are processed efficiently to yield physiological levels of the active guide strand [29], [30], [31]. We therefore sought to design a series of primary microRNA (pri-miRNA) mimics targeting mutant *atxn7* that incorporated single nucleotide mismatches at successive positions tiled across the active strand of a pri-miRNA. Multiple pri-miRNA designs were generated (pri-miRNA hairpin structures shown in Figure 6A), all based on the miR-122 pri-miRNA which is known to yield homogenous mature miRNA species following Drosha and Dicer processing [32]. In this study miR-P16 was found to be the most effective pri-miRNA, demonstrating highly effective mutation-specific *atxn7* silencing in both the hemizygous situation and in the heterozygous cellular model (Figures 6B and Figure S3). Indeed, our data shows that miR-P16 provided comparable levels of knockdown against the mutant and wild-type *atxn7* transcripts as was found for the shR-P16 hairpin, with almost complete ablation of mutant *atxn7* expression (to less than 10% of control levels) with a minimal effect on the wild-type target (retaining 75% expression; Figure 6B). This corroborates the earlier data with respect to the position of the single nucleotide mismatch within the shRNA guide strand for optimal silencing of the *atxn7* mutant transcript. In addition, an siRNA with the mismatch as position 16 (siR-P16), shows discrimination (levels of target remaining are 55% and 30% of the wild-type and mutant respectively; Figure S5). This indicates that despite the additional processing required of the pri-miRNA, the same miR-P16 active strand sequence is likely to have been produced as that generated by shR-P16.

While toxicity due to interference with RNAi pathway functions is difficult to measure *in vitro* using standard cell toxicity assays, competition studies comparing the effects of shRNA vs miRNA based hairpins can be used to detect inhibitory effects on the post-transcriptional RNAi machinery [33]. Increasing concentrations of shR-P16 leads to a reduction in knockdown efficiency of the reporter gene GFP by a GFP specific hairpin (Figure 6C). The addition of 0.5 µg followed by 1 µg of shR-P16 leads to nearly 20% reduction in silencing of GFP (p<0.03). In contrast, the corresponding miRNA hairpin (miR-P16) shows no such reduction in knockdown efficiency of GFP over the same concentration range.

## Discussion

Post-transcriptional RNAi-based gene silencing has potential for the targeted silencing of disease mutations. In this study we have systematically studied RNAi hairpin design and how hairpin guide strand anatomy might be optimised for discriminating single nucleotide mismatches in the *atxn7* gene. Exploiting a CAG mutation-linked SNP we investigated allele-specific silencing of short and full-length *atxn7* target sequences in both hemizygous and heterozygous model systems, the latter mimicking aspects of this dominant neurodegenerative disease. While analysis of short *atxn7* targets revealed that RNAi hairpins incorporating nucleotide mismatches 3′ to the central region were most selective for mutant *atxn7* silencing, this approach was not predictive of the most efficacious hairpin for full-length target silencing. The hemizygous and heterozygous full-length *atxn7* model systems both revealed the importance of hairpins with the nucleotide mismatch predicted to be incorporated at the 3′ position 16, and in the heterozygous system this shRNA yielded over 90% mutant silencing. Moreover, treatment with shR-P16 led to phenotype correction in a cellular SCA7 model. The accuracy of the position 16 mismatch was corroborated by subsequent analysis in which a pri-miRNA based on miR-122 containing position 16 nucleotide mismatches also generated highly efficient allele-specific *atxn7* silencing.

The use of short mRNA targets fused to luciferase reporters is an efficient strategy to identify optimal siRNA sequences for target silencing [34], [35]. Schwarz *et al*. [17] utilised this approach extensively to screen siRNAs for the targeted silencing of mutant *Htt* and *SOD1* genes and reported that nucleotide mismatches in the 3′ region of the siRNA guide strand could allow greater single nucleotide selectivity than those placed centrally, in particular mismatches at position 16. The present study used a similar approach in which a 60 nucleotide *atxn7* target sequence was fused to a luciferase reporter and systematically identified the importance of single nucleotide mismatches placed at specific positions 3′ to the central position 10, in particular position 15, for efficient mutation-specific silencing (Figure 2). However, the mismatch at position 16 failed to show mutant discrimination with strong silencing of both mutant and wild-type short target sequences. This supports the general findings of Schwarz *et al*. in revealing that the significance of the 3′ region for single nucleotide discrimination extends to expressed RNAi hairpins, however importantly this data was not predictive of the most efficacious shRNA targeting full-length mutant *atxn7*. That the shR-P15 was most discriminatory for silencing of a short *atxn7* target (and not position 16 as shown by Schwarz *et al.* 2006), could simply be due to sequence context and the nature of the nucleotide mismatch in this case. Despite extensive modifications designed to thermodynamically bias shRNA guide strand selection (Figure 1A) it is possible that strand selection by RISC may have affected shR-P15 knockdown efficiency [36], [37], however it is unlikely that this would have influenced the *degree* of discrimination.

In general target knockdown efficiency was diminished from the luciferase assay to the full-length target studies, and shR-P15 which had previously shown the highest level of mutant selectivity against the short target sequence failed to knockdown the full-length mutant target (Figures 3 and 4). However, in both hemizygous and heterozygous full-length target studies the shR-P16 showed strong mutant selectivity with minimal effect against the wild-type. The case of shR-P15 highlights the importance of screening full length target sequences. While shR-P15 showed good mutant selectivity against a short target sequence it was ineffective against a longer full length sequence, a finding likely to be explained by structural constraints imposed by the longer mRNA sequence, given that full-length mRNA secondary structure can influence the efficiency of RISC-associated cleavage [38]. Thus, the data suggest that screening of short mRNA targets might not be a reliable method for identifying optimal mutation-specific RNAi hairpins (or siRNAs) for *in vivo*/clinical use. To corroborate the shRNA data a second approach utilising a pri-miRNA hairpin sequence was investigated. The miR-122 backbone was selected for these experiments given that it has been demonstrated to yield homogenous guide strand sequences as opposed to the more heterogeneous products from miR-31 [32]. Particular effort was made to replicate the thermodynamic structure of miR-122 and wherever possible similar strength mismatches were maintained. Importantly miR-P16 (designed to place the nucleotide mismatch against the wild-type *atxn7* at the same position as was used in shR-P16) showed the greatest mutant selectivity in both full-length cell-based assays, with very similar mutant discrimination to that seen for the corresponding shRNA, corroborating the earlier data (Figure 6, and Figure S3). These data support the conclusion that the hairpins generated active guide strands with the mismatch at position 16. *In vivo* studies have shown that over expression of shRNA may lead to toxicity [30]. Although this phenomenon may be resolved by different shRNAs and/or reducing their transcription, expression of miRNA-based hairpins offer an attractive alternative [33], [39]. Our results suggest that use of miRNA shuttles may confer some advantage over the shRNA design. This is supported by our observation that that miR-P16 showed no apparent disruption of endogenous post-transcriptional silencing, which was in contrast to what was observed with the shR-P16 expression cassette.

The shR-P16 allowed phenotype correction in a cellular SCA7 model system, resulting in decreased levels of both mutant and wild-type ATXN7 aggregates. While the reduction in mutant aggregates correlated with a reduction in mutant protein expression, the decrease in wild-type aggregates correlated with an increase in the number of cells showing a dispersed pattern of wild-type ATXN7 protein expression. This is important for two reasons. First, some studies suggest that mutant aggregate formation is protective and thus inhibition of aggregate formation may have detrimental effects [40]. In the present study the prevention of large aggregate formation occurred together with reduction in the level of toxic mutant protein and thus is very likely to be beneficial. Second, it has been reported that mutant ATXN7 protein recruits the wild-type protein into intracellular aggregates [28] and thus pathogenesis in this polyQ disease may include an element of loss-of-function of the wild-type protein contributing to disease progression [41]. As a consequence of the allele-specific knockdown of the mutant *atxn7* gene we observed that while the expression level of wild-type ATXN7 protein was largely unchanged its localisation was restored to a non-aggregated, more uniform cellular distribution. It is likely that the function of the wild-type protein in transcriptional regulation [42]–[44] is inhibited by its sequestration into aggregates, and thus reversal of this loss-of-function may contribute to the efficacy of this therapeutic approach. Further studies in animal models and/or patient derived stem cells will be needed to test this hypothesis. While targeted silencing of the mutant allele is the most ideal therapeutic approach, two recent publications targeting the huntingtin protein in a non-allele specific manner demonstrate therapeutic benefits *in vivo* [45], [46]. This may well be relevant to other polyQ disorders such as SCA7, given the added technical challenge of allele-specific silencing. The critical questions are the level to which the specific wild-type protein is reduced in such a non-allele specific approach and to what extent such loss is likely to be tolerated in patients with a late onset neurodegenerative disease.

The identification of shRNA- and pri-miRNA-mismatched hairpins with high selectivity for mutant *atxn7* and the finding that mutant selectivity via targeting a weak nucleotide mismatch may have beneficial phenotypic effects through decreasing mutant ATXN7 aggregates and restoring wild-type protein to its native distribution pattern, suggests that selective RNAi hairpins may have therapeutic benefits for many of the currently untreatable polyQ disorders. Moreover, the identification of the highly selective pri-miRNA offers a hairpin structure likely to better tolerated [29]–[31] and more amenable for tissue-specific targeting in future *in vivo* studies. Given that SCA7 is a neurodegenerative disorder with a unique macular phenotype, this would provide an ideal model for testing the efficacy of mutant-specific silencing in patients using an RNAi-based allele-specific silencing approach.

## Materials and Methods

### Plasmid construction

Construction of shRNA-expressing vectors has been previously described [47]. All expected shRNA duplexes targeting the G>A SNP in *atxn7* are indicated in Figure 1A and Figure S1A. Construction of miRNA-based vectors has been described [32]. All expected miRNA duplexes targeting the G>A SNP in *atxn7* are indicated in Figure 6. Short target sequences of the *atxn7* transcript were cloned into psiCHECK (Promega) that expresses the *Renilla* luciferase reporter gene (Figure 1B). Seventy-four bp of annealed DNA oligonucleotides harbouring each of the target sequences and *Not*I and *XhoI* sticky ends were ligated into the psiCHECK plasmids downstream of the *Renilla* reporter gene. Full-length *atxn7* cDNA constructs (wild-type (CAG)_10_ and mutant (CAG)_100_) were fused to the N-terminus of the reporter gene, enhanced green fluorescent protein (eGFP), kindly obtained from A Brice [28]. Mutant *atxn7* (CAG)_100_ (also obtained from A Brice) was modified to replace the G residue of the SNP (rs3774729) with the mutant A allele. Long oligonucleotides were annealed and subjected to an initial primer extension with *taq* polymerase; this was followed by a secondary primer extension to produce *Xho*I and *Hind*III sticky ends. The resultant 150 bp dsDNA cassette harbouring the A allele with *XhoI* and *Hind*III ends was cloned into the mutant vector (CAG)_100_ linearised with *XhoI* and *Hind*III. The generated (atx7-100-A-eGFP) A allele was confirmed by DNA sequencing. For the heterozygous assay, the mutant atx7-100-A-eGFP was modified to substitute the eGFP gene with the red fluorescent protein from pDsRED-monomer-N1 vector (Clontech). pDsRED-monomer-N1 was linearised with *HpaI* and *Age* and the resulting dsRED fragment was cloned into the atx7-100-A-eGFP vector which had been linearised with the same restriction endonucleases to remove the eGFP gene. The sequence of the mutant vector (atx7-100-A-DsRED) was confirmed. pDsRED-monomer-N1 was chosen because it does not result in aggregate formation as other DsRED protein has been shown to, therefore any aggregate formation is as a result of the fused target protein [48]. siRNAs were a kind gift from Novartis.

### Cell culture and transfections

HEK293 cells were cultured in Dulbecco's modified Eagle's medium supplemented with 10% fetal calf serum, L-glutamine (4 mM), penicillin (50 IU/ml), and streptomycin (50 mg/ml). Plasmid transfections using the luciferase targets were performed using Lipofectamine 2000 (Invitrogen), according to manufacturer's instructions, in 24 well plates with a total of 1 µg of DNA in a 1∶1 ratio of target∶shRNA vector. Transfections for the hemizygous full-length target fluorescence assays were performed using jetPEI (Polyplus) with an N/P ratio of 5, using a total of 2 µg of DNA in a 1∶1 ratio of target∶shRNA vector. The use of HEK293 cells for this assay was validated by the fact that endogenous levels of *atxn7* are insignificant in comparison to the expression of polymerase II driven expression targets, with a greater than 500 fold increase in *atxn7* transcript in transfected cells compared to untransfected cells (data not shown). siRNA transfections were carried out with a final concentration of 50 nM in 24 well plates, using a total of 1 µg of target DNA, and transfected with 2 µl of jetPEI, in an attempt to replicate those conditions described above. Heterozygous assays using shRNAs were performed as above except using 6-well plates and a total of 6 µg of DNA. In cases where target vectors were transfected separately for comparison in the heterozygous experiments, pTZU6+1 was included to equate the total amount of DNA transfected to that in the co-transfections of both targets. Transfections of miRNAs in the heterozygous conditions were performed as above except using a ratio of 2∶1 of target to vector in order to compensate for saturation of the polymerase II transcription machinery. Competition assays were performed by transfecting 50 ng of GFP target vector (pGFPmax, Amaxa) and 50 ng of shR-E19. A total of 1 µg of additional plasmid DNA was transfected in the following manner; for the shRNA assay: 1 µg pU6+1; or 0.5 µg pU6+1 and 0.5 µg shR-P16; or 1 µg shR-P16. GFP expression in each was normalised to shR-NS with 1 µg pU6+1. For the miRNA assay the following amounts of DNA were added: 1 µg pCI_neo; or 0.5 µg pCI_neo and 0.5 µg miR-P16; or 1 µg miR-P16. Transfections were performed in a 24 well plate using the same number of cells and N/P ratio as described above.

### Luciferase assay

The activities of *Renilla* and firefly luciferase were measured 48 hours after transfection with the dual luciferase assay kit (Promega, WI) and using the Veritas dual injection luminometer (Turner BioSystems, Sunnyvale, CA).

### Fluorescence imaging and quantification

Fluorescence was quantified using the *Optima* software program on an Optima FLUOSTAR fluorimeter. All hemizygous quantitative experiments were performed by normalizing to 100 µg/ml of protein using the Micro BCA Protein Kit (Pierce, USA) extracted 48 hours after transfection. Average values are shown from experiments in biological triplicate with values from indicated U6/shRNA expression plasmids normalized to a miss-targeted shRNA expression plasmid set at 1. This was compared to an empty U6 vector to ensure that the non-specific sequence had no effect on levels of target expression (data not shown). Error bars indicate variation between experiments as the standard deviation of the mean. For the hemizygous experiments and the competition assay, equivalent quantities of mock-transfected cells seeded in 24-well plates were used to subtract the background fluorescence. Both eGFP and DsRed have an excitation wavelength 489 nm and 556 nm respectively; and emission of 508 nm and 586 nm respectively (Clontech). In measuring the fluorescence dual assay of eGFP and DsRed an overlap can occur and influence the fluorescent measurements of both. In order to avoid the overlap of green and red fluorescence in a dual heterozygous assay, modifications were made to the previously described assay. Firstly the excitation and emission wavelengths for both proteins were adjusted to create minimal overlap, such that eGFP and DsRed were read at excitation wavelengths of 485 nm and 544 nm respectively; and emissions of 520 nm and 590 nm respectively. Secondly because the overlap cannot be completely removed, equivalent quantities of cells transfected with 0.5 ug atxn7-10-G-eGFP +1.5 µg of pTZU6+1 were used as the background values whilst reading through the red channel. Conversely, equivalent quantities of cells transfected with 0.5 ug atxn7-100-A-DsRED +1.5 µg of pTZU6+1 were used as the background values whilst reading through the green channel. Protein was extracted after 72 hours and concentrations of 200 µg/ml of protein were assayed to maximise the level of reporter gene expression. shR-E19 is a U6/shRNA expression plasmid targeting eGFP, a kind gift from Y Yokobayashi [49]. Representative images shown were taken on a ZEISS confocal microscope and the data analyzed by the ZEISS LSM 4.1 software.

### Aggregate counting

Aggregate-containing cells were counted as previously shown [50] with the following modifications. Cells were co-transfected in triplicate as described in the heterozygous assay. Non-overlapping frames of live cells from each well were captured on an AxioVision Inc. microscope using the AxioVision 4.6.3 software after 72 hours. The sum of the total number of cells in three frames was calculated using a 40× objective. Cells with aggregates and those with dispersed patterns of expression were counted as a total of the three different frames of live cells. This was performed in triplicate with the mean representing the average number of that biological triplicate. It is noted that previous studies using the tagged vectors had shown that the reporter genes had no effect on the localisation and expression pattern of the ATXN7 protein [28].

### Statistical Analysis

Statistical analysis utilized two-tailed t-tests, unless otherwise stated. Statistical differences were considered significant when the two-tailed p value<0.05 using a paired t-test.

## Supporting Information

Figure S1 Analysis of series of double mismatched shRNA guide sequences targeting the G>A SNP in atxn7. (A) Relative levels of Renilla luciferase (hRluc) expression normalized to firefly luciferase (hFluc) expression measuring both the wild-type (atx7-G-luc) and mutant (atx7-A-luc) target knockdown. (B) Quantitation of fluorescence in HEK293 cells transfected with wild-type (atx7-10-G-eGFP) or mutant (atx7-100-A-eGFP) expression plasmids and the indicated shRNA. The shR-E19 hairpin was pE19; a published U6/shRNA expression plasmid targeting eGFP (37). Each experiment was performed in triplicate and the data is relative to that measured using a non-specific shRNA, NS. Average±standard deviation is shown. Statistically significant differences (p<0.05) between wild-type and mutant silencing are indicated by corresponding p values. Relative expression of wild-type and mutant targets is represented by red and blue bars respectively.
(1.87 MB EPS)

Figure S2 Additional mismatches to the atxn7 SNP. A) Convention for the diagram follows Schwarz et al. (2006) (16). Sequences of predicted mismatch guide strands (underlined) processed from an shRNA format indicating the site of the primary mismatch to the G allele of the wild-type atxn7 target. Functional asymmetry was induced by creating GU mis-pairings in the 3′ end of the anti-guide strand; these are highlighted in bold. The shRNAs were labelled according to the position of the primary mismatch relative to the 5′ end of the guide strand; followed by any additional mismatches. The nomenclature is based on a 21bp guide strand after cleavage of the anti-sense strand by Dicer. (B) Full-length hemizygous assay. Quantitation of fluorescence in HEK293 cells transfected with wild-type (atx7-10-G-eGFP) or mutant (atx7-100-A-eGFP) expression plasmids and the indicated shRNA. Each experiment was performed in triplicate and the data is relative to that measured using a non-specific shRNA, NS. The anti-eGFP shRNA used was pE19; a published U6/shRNA expression plasmid targeting eGFP (37). Average±standard deviation is shown. Wild-type and mutant targets are represented by red and blue bars respectively.
(1.34 MB EPS)

Figure S3 Pri-miRNAs targeting the atxn7 G>A SNP in a full-length hemizygous assay. Quantitation of fluorescence in HEK293 cells transfected with wild-type (atx7-10-G-eGFP) or mutant (atx7-100-A-eGFP) expression plasmids and the indicated shRNA and miRNA. Each experiment was performed in triplicate and the shRNA data is relative to that measured using a non-specific shRNA, NS, while the miRNA data is relative to that measured using a non-specific miRNA, NS. The shR-E19 hairpin was pE19; a published U6/shRNA expression plasmid targeting eGFP (37). Average±standard deviation is shown. Statistically significant differences (p<0.05) between wild-type and mutant silencing are indicated by corresponding p values. Wild-type and mutant targets are represented by red and blue bars respectively.
(9.64 MB EPS)

Figure S4 Knockdown of targets in a hemizygous assay using shRNAs. Quantification of fluorescence in HEK293 cells transfected with mutant atx7-100-G-eGFP or mutant atx7-100-A-DsRED expression plasmids represented by blue or red bars respectively. The indicated hairpin is shown. Each experiment was performed in triplicate and the data is relative to that measured using a non-specific shRNA, NS. The shR-E19 hairpin was pE19; a published U6/shRNA expression plasmid targeting eGFP (37). Average±standard deviation is shown. Statistically significant differences (p<0.05) between wild-type and mutant silencing are indicated.
(0.98 MB EPS)

Figure S5 Comparison of siR-P16 and shR-P16 targeting the atxn7 G>A SNP in a full-length hemizygous assay. Quantitation of fluorescence in HEK293 cells transfected with wild-type (atx7-10-G-eGFP) or mutant (atx7-100-A-eGFP) expression plasmids and the indicated small RNA is shown. Each experiment was performed in triplicate and the data is relative to that measured using a non-specific shRNA, NS or siRNA, NS. The siR-eGFP is an siRNA with specificity for eGFP. Average±standard deviation is shown. Statistically significant differences (p<0.05) between wild-type and mutant silencing are indicated by corresponding p values.
(0.83 MB EPS)
